# Determinants of Venous Thromboembolism among Hospitalizations of US Adults: A Multilevel Analysis

**DOI:** 10.1371/journal.pone.0123842

**Published:** 2015-04-16

**Authors:** James Tsai, Althea M. Grant, Michele G. Beckman, Scott D. Grosse, Hussain R. Yusuf, Lisa C. Richardson

**Affiliations:** Division of Blood Disorders, National Center on Birth Defects and Developmental Disabilities Centers for Disease Control and Prevention, Atlanta, Georgia, United States of America; College of Medicine, University of Ibadan, University College Hospital, Ibadan, Nigeria., NIGERIA

## Abstract

**Background:**

Venous thromboembolism (VTE) is a significant clinical and public health concern. We evaluated a variety of multilevel factors—demographics, clinical and insurance status, preexisting comorbid conditions, and hospital characteristics—for VTE diagnosis among hospitalizations of US adults.

**Methods:**

We generated adjusted odds ratios with 95% confidence intervals (CIs) and determined sources of outcome variation by conducting multilevel logistic regression analysis of data from the 2011 Nationwide Inpatient Sample that included 6,710,066 hospitalizations of US adults nested within 1,039 hospitals.

**Results:**

Among hospitalizations of adults, age, sex, race or ethnicity, total days of hospital stay, status of health insurance, and operating room procedure were important determinants of VTE diagnosis; each of the following preexisting comorbid conditions—acquired immune deficiency syndrome, anemia, arthritis, congestive heart failure, coagulopathy, hypertension, lymphoma, metastatic cancer, other neurological disorders, obesity, paralysis, pulmonary circulation disorders, renal failure, solid tumor without metastasis, and weight loss—was associated independently with 1.04 (95% CI: 1.02−1.06) to 2.91 (95% CI: 2.81−3.00) times increased likelihood of VTE diagnosis than among hospitalizations of adults without any of these corresponding conditions. The presence of 2 or more of such conditions was associated a 180%−450% increased likelihood of a VTE diagnosis. Hospitalizations of adults who were treated in urban hospitals were associated with a 14%−15% increased likelihood of having a VTE diagnosis than those treated in rural hospitals. Approximately 7.4% of the total variation in VTE diagnosis occurred between hospitals.

**Conclusion:**

The presence of certain comorbidities and hospital contextual factors is associated with significantly elevated likelihood of VTE diagnosis among hospitalizations of adults. The findings of this study underscore the importance of clinical risk assessment and adherence to evidence-based clinical practice guidelines in preventing VTE, as well as the need to evaluate potential contextual factors that might modify the risk of VTE among hospitalized patients.

## Introduction

Venous thromboembolism (VTE), comprising deep vein thrombosis (DVT) and pulmonary embolism (PE), is a serious clinical and public health concern [1, 2]. VTE not only imposes a substantial socioeconomic burden on healthcare systems, but also is responsible for numerous debilitating health consequences, including morbidity, mortality, disability, and diminished health-related quality of life among affected individuals [1–3]. In the United States, based on the analyses of hospital discharge data for the years 2009−2010, diagnoses of VTE were recorded in as many as 770,000 hospitalizations of adults annually [4, 5]. Studies also have found that more than 70% of patients who experienced VTE events had VTE identified in a community or outpatient setting, and at least 30% of these outpatients with VTE had been hospitalized during the previous 3 months [6, 7]. While the classic Virchow’s triad describes vascular endothelial damage, stasis of blood flow, and hypercoagulability of blood as the three general components for the pathogenesis of venous thrombosis, advances in research have further identified many specific demographic (e.g., advanced age), biological (e.g., increased levels of prothrombin and fibrinogen), behavioral (e.g., smoking), environmental (e.g., long-distance travel), and health conditions-related risk factors (e.g., surgical and comorbid conditions) that show a simultaneous presence or a sequential interplay of transient and persistent risk factors in the development of VTE [8–10].

As an important health condition that potentially is preventable through implementation of thrombosis risk assessment and evidence-based clinical practice guidelines, VTE is especially a concern for hospitalized patients [11–13]. Previous studies have suggested hospital characteristics (e.g., hospital size, ownership, and teaching status) might influence health outcomes (e.g., short-term mortality and complications) among hospitalized patients [14, 15]. Currently, there is considerable interest among health care providers, researchers, and public health professionals to identify individual- and group-level risk factors for VTE, to discern “compositional effects” (e.g., effects of patient characteristics) and “contextual effects” (e.g., effects of socio-environmental or neighborhood characteristics) and to determine sources of variation (e.g., within group and between group) in VTE risk [11, 16, 17]. Such evidence is essential to identify accurately patients who might benefit from VTE prophylaxis and to inform prevention strategies for optimal care. Therefore, to strengthen the epidemiologic evidence and to fill the knowledge gap in this largely unexplored area of research, we evaluated multilevel determinants, comprising demographics, clinical and insurance status, preexisting comorbid conditions, and hospital characteristics, of VTE diagnosis among hospitalizations of US adults by analyzing data from the 2011 Nationwide Inpatient Sample (NIS).

## Methods

### Ethics Statement

All procedures involving human participants and confidentiality were reviewed and approved by the Research Ethics Review Board of the Agency for Healthcare Research and Quality (AHRQ). Patient records for the NIS were anonymized and de-identified prior to public release for analysis. Analysis of deidentified data from the NIS is exempt from federal regulations for the protection of human research participants.

### Data Source

The NIS is part of the Healthcare Cost and Utilization Project sponsored by the AHRQ [18]. It is the largest all-payer inpatient care public database in the United States, with data on 5−8 million annual unweighted hospitalizations of patients covered by Medicare, Medicaid, or private insurance, and the uninsured from about 1,000 community hospitals. The NIS sampling frame comprises non-federal, short-term, general and specialty hospitals, and long-term acute care facilities. Excluded from the NIS are short-term rehabilitation hospitals, long-term non-acute care hospitals, psychiatric hospitals, and alcoholism or chemical dependency treatment facilities. The NIS is designed to approximate a 20% stratified sample of U.S. community hospitals that include all hospitalizations in the sampled hospitals [18]. The 2011 inpatient core file contained data for 8,023,590 hospitalizations drawn from 1,049 hospitals in participating states that make up 97% of the population in the United States [19]. Details about the sampling methodology are described elsewhere [18]. We restricted our analysis to a sample of hospitalizations for adults 18 years of age and older (n = 6,834,910). After further excluding hospitalizations related to pregnancy, childbirth and puerperium and those with missing information for the specific potential determinants we examined (patient age, sex, and total days of hospital stay; insurance status of primary expected payer; and hospital bed size, location and teaching status, and ownership), a total of 6,710,066 hospitalizations of adults nested within 1,039 hospitals were used as the analytical sample.

### Outcome Variable

The NIS provides a maximum of 25 fields of diagnosis based on the *International Classification of Diseases*, *Ninth Revision*, *Clinical Modification* (*ICD-9-CM*) for each sampled hospitalization. We determined VTE diagnosis by the presence of *ICD-9-CM* codes 415.1x, 451.1x, 451.2, 451.8x, 451.9, 453.2, 453.4x, 453.8x, and 453.9 in any of the 25 fields of diagnosis.

### Multilevel Determinants of VTE Diagnosis

We assessed the potential determinants of VTE diagnosis at the levels of hospitalization and hospitals. Those variables were selected for inclusion because they are well-known risk factors for VTE or are relevant to VTE prevention.

#### Hospitalization level

We included demographics (i.e., age, sex, and race or ethnicity−10.1% unstated), clinical and insurance status (i.e., total days of hospital stay, insurance status of primary expected payer, and status of operating room procedure), and 29 preexisting comorbid conditions. The AHRQ 29-comorbidity index originally was developed in 1998 by Elixhauser and colleagues for risk adjustment in health outcome research using administrative data [20]. Previous research has demonstrated that the index can predict accurately health outcomes such as mortality for a general population and for hospitalizations of adults with a VTE diagnosis [4]. In the NIS, these 29 comorbidities are considered as coexisting medical conditions that are not related directly to the principal diagnosis or the main reason for admission, and are likely to have existed prior to the hospital stay [21]. Additionally, all *ICD-9-CM* procedure codes in the NIS are assigned to one of four broad categories of procedures—minor diagnostic, minor therapeutic, major diagnostic, and major therapeutic—according to the AHRQ Procedure Classes [22]. An operating room procedure was defined as having at least one major diagnostic or major therapeutic procedure during hospitalization [23].

#### Hospital level

We assessed the following hospital characteristics bed size (i.e., small, medium, and large), location and teaching status (i.e., rural, urban non-teaching, urban teaching), and ownership (i.e., “government, nonfederal,” “private, non-profit,” and “private, investor-own”) as potential hospital-level determinants. Approximately one-third of the hospitals in a given region, location, and teaching status combination were classified into categories of small, medium, or large bed size by the NIS [19]. Because rural teaching hospitals were rare and accounted for less than 2% of all hospitals, rural status within the category of “location and teaching” was not classified further by teaching status in the NIS [19].

### Statistical Analysis

We calculated the percentage distribution for and rate of VTE diagnosis among hospitalizations of adults by age, sex, race or ethnicity, total days of hospital stay, insurance status of primary expected payer, and operating room procedure. We also estimated the number and percentage distributions of hospitals by bed size, location and teaching status, and ownership. Because the outcome of interest (i.e., VTE diagnosis) was correlated among each group of hospitalizations of adults who were treated within the same hospitals, we applied a multilevel modeling approach to address clustered data in which analytical units (e.g., individual hospitalizations of adults) were nested within other units of interest (e.g., hospitals) [16]. To evaluate the associations between covariates and outcome (fixed effects) and to determine the variation in outcome (i.e., probabilities of VTE diagnosis) between hospitals (random effects) while accounting for clustered data structure, we specified four 2-level logistic regression models with fixed slopes and a random intercept for each model [16]. More specifically, we constructed model 0, an unconditional empty model with no covariate, to divulge the probabilities of VTE diagnosis between hospitals. Model 1 comprised demographic characteristics, clinical and insurance status (i.e., age, sex, race or ethnicity, total days of hospital stay, insurance status of primary expected payer, and operating room procedure) as individual hospitalization level determinants (*P* < 0.05). Model 2 comprised all model 1 determinants and significant preexisting comorbid conditions (*P* < 0.05). Model 3 comprised model 2 determinants and hospital level determinant (i.e., location and teaching status) (*P* < 0.05). We employed a backward elimination procedure of stepwise regression to remove any predictor with the highest *P* ≥ 0.05 for individual *t*-test of null hypothesis *β* = 0. We repeated the same procedure until *P* < 0.05 for all predictors in models 1−3. The adjusted odds ratios (aORs) with 95% confidence intervals (CIs) were generated to measure the strength of the associations. The intercept variance and intraclass correlation coefficient

$$\left[ICC\;=\;\frac{Intercept\;variance}{\;Intercept\;variance\;+\;residual\;variance}\;=\;\frac{\sigma^{2}\;(variance\;between\;hospitals)}{\;\sigma^{2}(variance\;between\;hospitals)+\sigma^{2}\;(variance\;within\;hospitals)}\right]$$

were calculated to assess between- and within-hospitals variation in VTE diagnosis.[24] We performed weighted analyses to present nationally representative percentage estimates (i.e., frequency distribution and rate of VTE) using Complex Samples for Survey Analysis with SPSS 21 (*IBM Corp*) and STATA 13 (*StataCorp LP*) to account for the complex sampling design. We conducted multilevel modeling using standard procedure for generalized linear mixed models with robust variance estimation [16, 25].

## Results

### Characteristics of Hospitalizations and Hospitals

**Fig 1:**
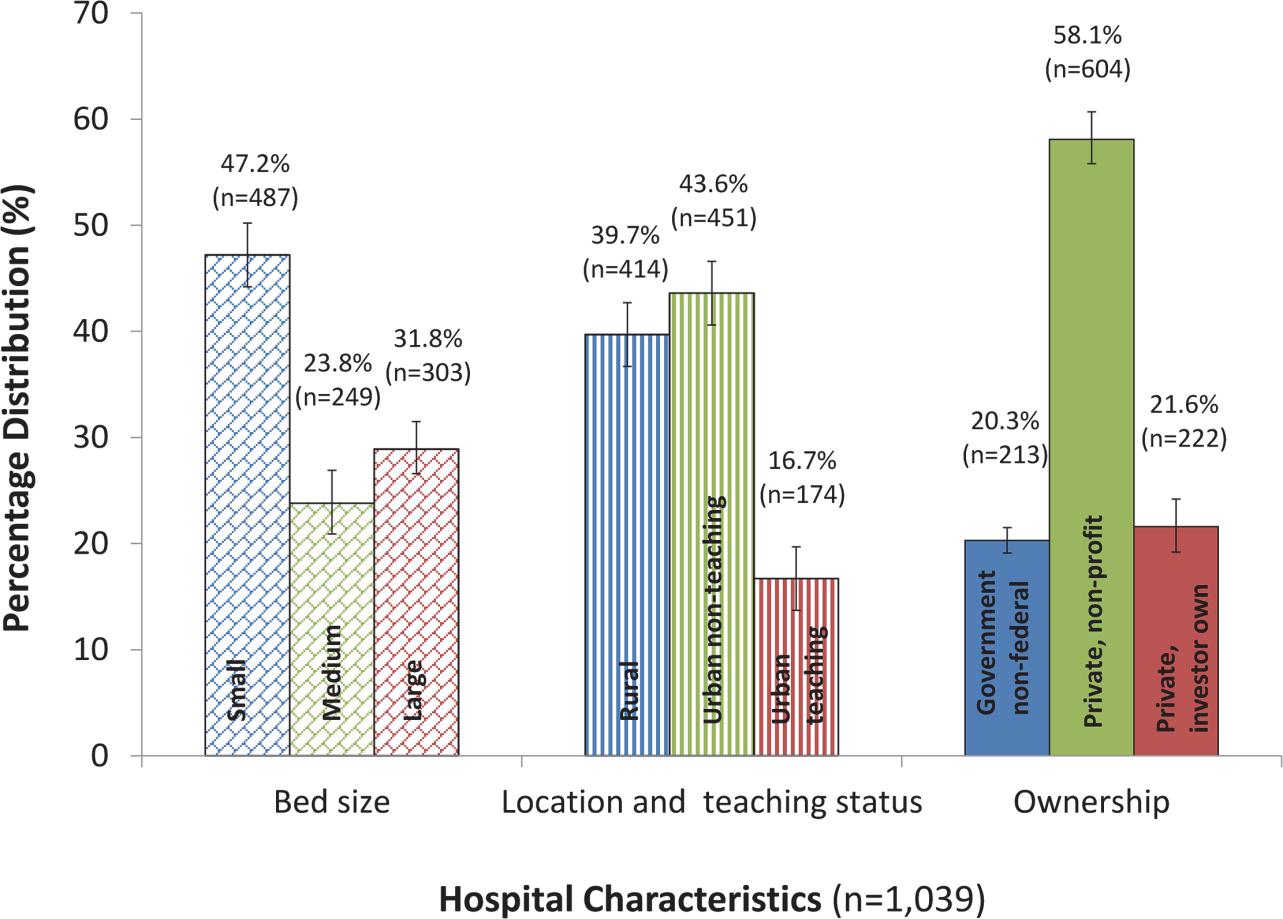
Number and percentage distributions of hospitals (n = 1,039) by hospital characteristics, NIS, 2011.

Table 1 shows that large percentages of all hospitalizations were of adults who were 50−79 years of ages (47.9%), were female (59.6%), were White (62.3%), had less than 7 total days of hospital stay (81.2%), had Medicare as the primary expected payer (46.9%), and had no operating room procedures of any kind (36.0%) (Table 1). In 2011, the rate of VTE diagnosis was 2.4% among hospitalizations of adults overall and varied significantly by age, sex, race or ethnicity, total days of hospital stay, insurance status of primary expected payer, and status of operating room procedure (*P* < 0.001). In addition, high rates of VTE diagnosis were observed among subgroups of hospitalizations of adults who were 80 years of age or older (3.0%), were male (2.9%), were Black (2.7%), had at least 7 total days of hospital stay (5.7%), had Medicare insurance as primary expected payer (2.9%), and had no operating room procedures of any kind (2.7%) (Table 1). Large percentages of the 1,039 hospitals in the sample were small bed size (42.2%); urban non-teaching (43.6%); or private, nonprofit (58.1%) (Fig 1).

**Table 1:** Percentage distribution and rate of VTE diagnosis among hospitalizations of US adults by demographic characteristics, clinical and insurance status, NIS, 2011.

Demographic characteristics, clinical and insurance status	Sample distribution			Rate of diagnosis of VTE		
	n^a^	%^b^	CI^c^	%	CI	*P-Value* ^d^
**Overall**	6,710,066	100	−	2.4	2.4−2.5	
**Age (years)**						*<0*.*001*
18−49	2,305,943	34.3	33.5−35.2	1.5	1.4−1.5	
50−79	3,210,405	47.9	47.3−48.4	2.9	2.8−3.0	
≥ 80	1,193,718	17.8	17.3−18.4	3.0	2.9−3.0	
**Sex**						*<0*.*001*
Male	2,708,723	40.4	39.9−40.9	2.9	2.8−2.9	
Female	4,001,343	59.6	59.1−60.1	2.1	2.1−2.2	
**Race or ethnicity** ^e^						*<0*.*001*
White	4,176,630	62.3	59.7−64.7	2.5	2.4−2.6	
Black	929,079	13.9	12.6−15.3	2.7	2.6−2.9	
Hispanic	641,244	9.6	8.2−11.3	1.7	1.5−1.8	
Other^f^	338,916	5.0	4.4−5.8	1.7	1.6−1.9	
Not stated	624,197	9.2	7.1−11.8	2.5	2.3−2.7	
**Total days of hospital stay**						*<0*.*001*
< 7 days	5,453,256	81.2	80.7−81.7	1.7	1.6−1.7	
≥ 7 days	1,256,810	18.8	18.3−19.3	5.7	5.5−5.8	
**Primary expected payer**						*<0*.*001*
Medicare	3,142,530	46.9	45.9−47.8	2.9	2.8−3.0	
Medicaid	1,003,379	15.0	14.1−15.9	1.6	1.5−1.7	
Private including HMO	1,972,074	29.3	28.3−30.4	2.2	2.1−2.2	
Self-pay or other payers	592,083	8.8	8.2−9.4	2.1	2.0−2.1	
**Operating room procedure**						*<0*.*001*
No procedure of any kind	2,404,657	36.0	34.9−37.1	2.7	2.6−2.8	
Non-operating room procedure^g^	2,328,118	34.6	33.8−35.5	2.3	2.2−2.4	
Operating room procedure^h^	1,977,291	29.4	28.7−30.1	2.2	2.1−2.3	

a. Unweighted sample size.

b. Weighted percentage may not add-up exactly to 100.0%.

c. 95% confidence interval.

*d*. *P*-value for Pearson Chi-Square test.

e. May not be nationally representative because not all participating states collect race or ethnicity information.

f. Included “other race/ethnicity,” “Native American,” and “Asian or Pacific Islander.”

g. Had at least one minor diagnostic or minor therapeutic procedure (without any major diagnostic or major therapeutic procedure) during hospitalization.

h. Had at least one major diagnostic or major therapeutic procedure during hospitalization.

### Measures of Fixed-Effect Association

**Fig 2:**
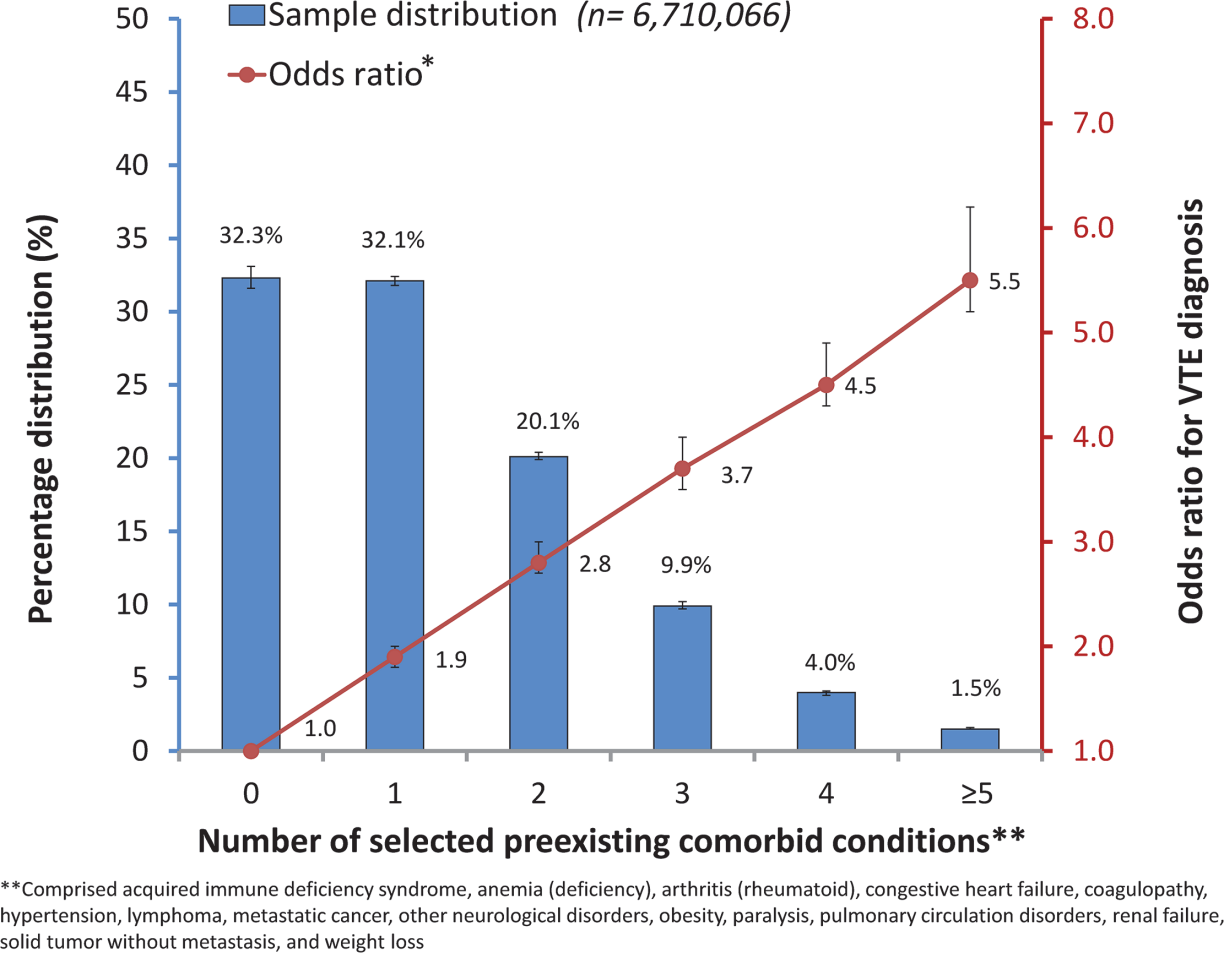
Percentage distribution of hospitalizations of US adults and odds ratios for VTE diagnosis by number of selected preexisting comorbid conditions, NIS, 2011.

The results of fixed-effect association based on three separate regression models are presented in Table 2. When compared with the corresponding subgroups of hospitalizations of adults who were 18−49 years of age, were female, were White, had less than 7 total days of stay, had Medicaid insurance as primary expected payer, or had an operating room procedure, subgroups of hospitalizations of adults who were 50−79 years of age (aOR = 1.50; 95% CI: 1.45−1.56), were 80 years of age or older (aOR = 1.55; 95% CI: 1.51−1.60), were male (aOR = 1.19; 95% CI: 1.17−1.20), were Black (aOR = 1.19; 95% CI: 1.17−1.22), had at least 7 total days of hospital stay (aOR = 3.48; 95% CI: 3.38−3.59), had Medicare insurance (aOR = 1.20; 95% CI: 1.17−1.24), had private including HMO insurance (aOR = 1.28; 95% CI: 1.23−1.32), had self-pay or other payers insurance (aOR = 1.15; 95% CI: 1.11−1.20), or had no procedure of any kind (aOR = 1.46; 95% CI: 1.40−1.53) were each positively and independently associated with a VTE diagnosis (model 1). By simultaneously adjusting for the significant preexisting comorbid conditions (*P* < 0.05) and determinants presented in model 1, each subgroup of hospitalizations of adults with acquired immune deficiency syndrome (AIDS), anemia, arthritis, congestive heart failure, coagulopathy, hypertension, lymphoma, metastatic cancer, other neurological disorders, obesity, paralysis, pulmonary circulation disorders, renal failure, solid tumor without metastasis, or weight loss was associated positively and independently with an increased likelihood (aORs ranged from 1.04 [95% CI: 1.02−1.06] to 2.91 [95% CI: 2.81−3.00]) of VTE diagnosis, compared with those without any of the corresponding 15 preexisting comorbid conditions (model 2). Upon further adjusting for hospital level determinant (i.e., hospital location and teaching status) (*P* < 0.05), hospitalizations of adults who were treated in “urban non-teaching” or “urban teaching” hospitals were associated with 1.14 (95% CI: 1.07−1.21) and 1.15 (95% CI: 1.08−1.23) times increased likelihoods of VTE diagnosis, respectively, when compared with a subgroup of hospitalizations of adults who were treated in rural hospitals (model 3). The patterns and strengths of association of demographic characteristics and clinical and insurance status with VTE diagnosis did not change appreciably in models 2 and 3. Fig 2 illustrates that approximately two of three hospitalizations of adults had at least 1 of the 15 preexisting comorbid conditions that were associated with a VTE diagnosis, and the cumulative number of these diseases corresponded to a 1.9- to 5.5-fold increased likelihood of VTE diagnosis (*P* < 0.001 for linear trend).

**Table 2:** Adjusted odds ratios for VTE diagnosis among hospitalizations of US adults by multilevel determinants, NIS, 2011.

Multilevel determinants^a^		VTE diagnosis					
		Model 1^b^		Model 2^c^		Model 3^d^	
	n^e^	OR^f^	95% CI^g^	OR	95% CI	OR	95% CI
**INDIVIDUAL HOSPITALIZATION LEVEL**	6,710,066						
**Age (years)**							
50−79 *(vs 18−49)*	3,210,405	1.50	1.45−1.56	1.38	1.34−1.42	1.38	1.34−1.42
≥ 80 *(vs 18−49)*	1,193,718	1.55	1.51−1.60	1.32	1.27−1.36	1.32	1.27−1.36
**Sex**							
Male *(vs Female)*	2,708,723	1.19	1.17−1.20	1.21	1.19−1.23	1.21	1.19−1.23
**Race or ethnicity**							
Black *(vs White)*	929,079	1.19	1.17−1.22	1.16	1.14−1.18	1.16	1.13−1.18
Hispanic *(vs White)*	641,244	0.78	0.75−0.81	0.79	0.77−0.82	0.79	0.77−0.82
Other^h^ *(vs White)*	338,916	0.78	0.74−0.83	0.79	0.74−0.84	0.79	0.74−0.84
Not stated *(vs White)*	624,197	0.91	0.85−0.98	0.93	0.88−1.00	0.94	0.88−1.00
**Total days of hospital stay**							
≥ 7 days *(vs < 7 days)*	1,256,810	3.48	3.38−3.59	3.00	2.91−3.08	3.00	2.91−3.08
**Primary expected payer**							
Medicare *(vs Medicaid)*	3,142,530	1.20	1.17−1.24	1.16	1.13−1.20	1.16	1.13−1.20
Private including HMO *(vs Medicaid)*	1,972,074	1.28	1.23−1.32	1.27	1.23−1.31	1.27	1.23−1.31
Self-pay or other payers *(vs Medicaid)*	592,083	1.15	1.11−1.20	1.21	1.16−1.26	1.21	1.16−1.26
**Operating room procedure**							
Non-operating room procedure^i^ *(vs operating room procedure* ^j^ *)*	2,328,118	1.02	0.99−1.06	0.97	0.93−1.00	0.97	0.93−1.00
No procedure of any kind *(vs operating room procedure)*	2,404,657	1.46	1.40−1.53	1.51	1.44−1.57	1.51	1.45−1.57
**Acquired immune deficiency syndrome**	15,934	− ^k^	−	1.33	1.22−1.45	1.33	1.22−1.45
**Alcohol abuse**	295,743	−	−	0.69	0.66−0.72	0.69	0.66−0.72
**Anemia** *(deficiency)*	1,172,235	−	−	1.26	1.24−1.28	1.26	1.24−1.28
**Arthritis** *(rheumatoid)/collagen vascular diseases*	179,371	−	−	1.24	1.21−1.28	1.24	1.21−1.28
Chronic blood loss anemia	159,605	−	−	− ^g^	−	−	−
**Congestive heart failure**	564,645	−	−	1.08	1.06−1.10	1.08	1.06−1.10
Chronic pulmonary disease	1,197,654	−	−	−	−	−	−
**Coagulopathy**	308,968	−	−	1.55	1.51−1.58	1.55	1.51−1.58
Depression	720,809	−	−	−	−	−	−
**Diabetes, uncomplicated**	1,254,886	−	−	0.85	0.83−0.86	0.85	0.83−0.86
Diabetes with chronic complications	301,405	−	−	−	−	−	−
Drug abuse	270,125	−	−	−	−	−	−
**Hypertension** *(uncomplicated & complicated)*	3,280,440	−	−	1.06	1.04−1.08	1.06	1.04−1.07
**Hypothyroidism**	749,975	−	−	0.96	0.94−0.98	0.96	0.94−0.98
**Liver disease**	189,225	−	−	0.84	0.81−0.87	0.84	0.81−0.87
**Lymphoma**	51,298	−	−	1.59	1.51−1.66	1.58	1.51−1.66
Fluid and electrolyte disorders	1,494,353	−	−	−	−	−	−
**Metastatic cancer**	143,200	−	−	2.91	2.81−3.00	2.91	2.81−3.00
**Other neurological disorders**	495,916	−	−	1.11	1.09−1.13	1.11	1.09−1.13
**Obesity**	733,122	−	−	1.39	1.36−1.42	1.39	1.36−1.42
**Paralysis**	160,997	−	−	1.24	1.20−1.28	1.24	1.20−1.28
**Peripheral vascular disorders**	393,677	−	−	0.83	0.81−0.85	0.83	0.81−0.85
**Psychoses**	305,283	−	−	0.97	0.94−1.00	0.97	0.94−1.00
**Pulmonary circulation disorders** ^l^	114,931	−	−	2.06	1.98−2.13	2.06	1.98−2.13
**Renal failure**	786,447	−	−	1.04	1.02−1.06	1.04	1.02−1.06
**Solid tumor without metastasis**	132,594	−	−	2.23	2.17−2.30	2.23	2.17−2.30
Peptic ulcer disease excluding bleeding	2,140	−	−	−	−	−	−
Valvular disease	237,468	−	−	−	−	−	−
**Weight loss**	322,748	−	−	1.41	1.38−1.44	1.41	1.38−1.44
**HOSPITAL LEVEL**							
Bed size							
Medium *(vs Small)*	1,600,501	−	−	−	−	−	−
Large *(vs Small)*	4,275,498	−	−	−	−	−	−
**Location and teaching status**							
Urban non-teaching *(vs Rural)*	2,843,551	−	−	−	−	1.14	1.07−1.21
Urban teaching *(vs Rural)*	3,107,244	−	−	−	−	1.15	1.08−1.23
Ownership							
Private, non-profit *(vs Gov*, *non-federal)*	5,038,418	−	−	−	−	−	−
Private, investor own *(vs Gov*, *non-federal)*	965,283	−	−	−	−	−	−
**Variance component**	**Model 0** ^m^	**Model 1**		**Model 2**		**Model 3**	
**Intercept variance**	0.261*	0.168*		0.136*		0.133*	
**Intraclass correlation coefficient** *(%)*	7.4	4.9		4.0		3.9	

a. Determinants are in bold.

b. Generalized linear mixed model that adjusted for age, sex, race or ethnicity, total days of hospital stay, primary expected payer, and operating room procedure.

c. Generalized linear mixed model that adjusted for all determinants in model 1 and significant preexisting comorbid conditions (i.e., acquired immune deficiency syndrome, alcohol abuse, anemia, arthritis, congestive heart failure, coagulopathy, diabetes, uncomplicated, hypertension, hypothyroidism, liver disease, lymphoma, metastatic cancer, other neurological disorders, obesity, paralysis, peripheral vascular disorders, psychoses, pulmonary circulation disorders, renal failure, solid tumor without metastasis, and weight loss).

d. Generalized linear mixed model that adjusted for all determinants in model 2 and hospital location and teaching status.

e. Unweighted sample sizes for non-referent subgroups are shown. Referent groups were hospitalizations of adults without corresponding conditions (referent subgroups n = 6,710,066—n for non-referent subgroups).

f. Odds ratio.

g. Confidence interval.

h. Included “other race/ethnicity,” “Native American,” and “Asian or Pacific Islander.”

i. Had at least one minor diagnostic or minor therapeutic procedure (without any major diagnostic or major therapeutic procedure) during hospitalization.

j. Had at least one major diagnostic or major therapeutic procedure during hospitalization.

k. Eliminated variable not in the final model.

l. Hospitalizations with PE and without other diseases in the category were classified as having negative status for the disorder.

m. Unconditional, empty model with no covariate.

*P < 0.001.

### Measures of Random-Effect Variation

The estimate of intercept variance suggested an appreciable variation in probability of VTE diagnosis occurred between hospitals. The value of ICC indicated that approximately 7.4% of the total variation or differences in probability of VTE diagnosis occurred between hospitals and might have been attributable to potential contextual factors of the hospitals (model 0).

## Discussion

By analyzing a large sample of 6.7 million hospitalizations of adults nested within 1,039 hospitals in the United States from the 2011 NIS, our study expanded existing research through evaluation of both compositional effects from individual hospitalizations and contextual effects from hospitals on VTE diagnosis in the United States. The simultaneous effects of multilevel determinants of VTE encompassing demographic, clinical and insurance status, preexisting comorbid conditions, and hospital characteristics have not been characterized and reported in the past. Our results showed that, of all hospitalizations of US adults, 64.4% had at least 1 and 35.6% had at least 2 of the 15 preexisting comorbid conditions (AIDS, anemia, arthritis, congestive heart failure, coagulopathy, hypertension, lymphoma, metastatic cancer, other neurological disorders, obesity, paralysis, pulmonary circulation disorders, renal failure, solid tumor without metastasis, and weight loss). When compared with hospitalizations of adults without the corresponding conditions, the presence of these preexisting comorbid conditions showed a 4%−191% increased likelihood of a VTE diagnosis; the presence of 2 or more of such conditions was associated a 190%−450% increased likelihood of a VTE diagnosis. Moreover, approximately a 7.4% variation in VTE diagnosis could have been attributable to contextual factors of hospitals. For example, we found that hospitalizations of adults at urban hospitals had about a 14%−15% increased likelihood of a VTE diagnosis relative to those at rural hospitals, regardless of the hospital’s teaching status.

Our findings were consistent with existing evidence showing that factors such as having an advanced age, being male (e.g., for recurrent VTE), being Black, and having had a prolonged hospital stay were associated with an increased risk for VTE [26, 27]. We observed an increased likelihood of a VTE diagnosis when comparing hospitalizations of adults who had no procedure of any kind with those who had an operative room procedure. Despite the greater risk for VTE among surgical patients, previous research showed that medical patients were less likely to receive VTE prophylaxis and had more episodes of VTE and higher rates of pulmonary embolism and death [7, 28–31]. We postulated that hospitalizations of adults with no procedure of any kind included mostly medical patients. Differences in the clinical practice of VTE prophylaxis among clinicians in healthcare settings for patients with various health conditions may affect VTE risk. In addition, some preexisting comorbid conditions (e.g., rheumatoid arthritis, congestive heart failure, pulmonary circulation disorders, obesity, hypertension, and cancer with or without metastasis) have been identified as risk factors for VTE in previous research [32–36]. Diseases or conditions that perhaps had attracted less prominent attention for their roles in VTE development included iron deficiency anemia, AIDS or human immunodeficiency virus infection, coagulopathy, neurological disorders, paralysis, renal failure, and weight loss [37–44]. In general, the coexistence of two or more conditions can affect other disease or health outcome through the etiological mechanisms of direct causation, associated risk factors, heterogeneity, and independence [45]. The use of our 15 identified preexisting comorbid conditions for VTE risk assessment and prediction might be explored and validated further in future studies.

Hospitals in which people receive care can influence health outcomes via availability and accessibility of health services, as well as prevailing practices and attitudes towards compliance with clinical guidelines and adherence to therapies. The exact reason for an elevated likelihood of VTE diagnosis in hospitalizations at urban hospitals compared with those at rural hospitals was unclear. It could be attributable to the increased availability, accessibility, and utilization of diagnostic services including computed tomography and magnetic resonance imaging, as well as concentration of patients with more complicated conditions. The evidence and effects of significant variation in VTE diagnosis between hospitals were consistent with typical neighborhood effects on health outcomes [46]. Earlier studies have suggested that hospital ownership and teaching status were not associated consistently with patient outcomes even though differences in disease-specific populations may exist [47, 48]. When comparing hospitalizations with Medicaid insurance as the primary expected payer, we observed an increased likelihood of VTE diagnosis among hospitalizations of adults with Medicare; private insurance, including an HMO; or self-pay insurance. Additional research is needed to discern the effect of a higher level structure defined by hospitals or alternative contextual boundaries (e.g., type of insurance payer, team and institutional support, practice, policy, and education) that might shape the environments and processes of healthcare services.

Our results were based on a cross-sectional study which is not designed to establish a cause-effect relationship. The analytical units of the study were hospitalizations and hospitals. The rate of VTE diagnosis among hospitalizations did not necessarily reflect rates per patients, as individual patients could have had multiple hospitalizations. Because the NIS did not identify multiple hospitalizations for individual patients, patient-level of analysis could not be performed. In addition, our study had several other limitations. First, we could not determine if a VTE event had been present on admission or occurred during a hospital stay. Second, 9.2% hospitalizations of adults had missing information on race which might lead to potential misclassification; therefore, caution should be excised when interpreting results related to race. Third, some preexisting comorbid conditions (e.g., obesity and weight loss) were not defined uniformly and measured objectively. The accuracy of *ICD-9-CM* diagnostic codes can be affected by physician documentation; the availability, accessibility, and utilization of diagnostic tests; and reimbursement guidelines [49]. Fourth, the length of hospital stay could be a cause or an effect of having VTE, since patients who develop VTE prior to discharge might also have a longer duration of hospital stay due to the need to treat the VTE. As such, the total days of hospital stay was not necessarily an independent variable for predicting VTE risk in this study. Nevertheless, we did not identify appreciable differences in the associations between having a VTE diagnosis and other covariates upon removing the variable of total days of hospital stay from the regression models. Finally, although we evaluated several hospital characteristics (such as bed size, location and teaching status, and ownership), the variation in VTE diagnosis may have arisen from alternative higher level structures and other contextual factors such as availability and accessibility of advanced equipment and services that were not evaluated [46, 50].

The results from this study have several important clinical and public health implications. To date, a number of evidence-based clinical practice guidelines for VTE prevention using pharmacologic agents and mechanical devices have been developed and are available for various patient populations [12, 13]. Given that more than two-thirds of hospitalizations of adults might have been associated with one or more preexisting comorbid conditions that might predispose them to a heightened risk for VTE and that the concerns of suboptimal VTE risk assessment and prophylaxis for patients, our findings signify the importance of adherence to VTE prevention guidelines. Health care practitioners need to be informed of various risk factors for VTE risk among hospitalized patients in order to perform appropriate risk assessments to identify and provide counseling to those who might benefit from VTE prophylaxis. Our results also might increase the awareness of VTE among patients and healthcare practitioners and thereby contribute to appropriate decisions for prophylaxis. Patients should keep their doctors informed about their medical history and health conditions, discuss VTE prevention with their doctors during their hospital stay, and adhere to prescribed therapies. Finally, the effects and complexity of multilevel risk factors for VTE illustrate the need for comprehensive public health approaches and evidence-based research to identify multidisciplinary strategies that integrate science, clinical practice, and institutional support to prevent VTE among hospitalized patients.

In summary, the results of our study indicate that the presence of certain comorbidities and hospital contextual factors is associated with significantly elevated likelihood of VTE diagnosis among hospitalizations of adults. The findings of this study underscore the importance of clinical risk assessment and adherence to evidence-based clinical practice guidelines in preventing VTE, as well as the need to evaluate potential contextual factors that might modify the risk of VTE among hospitalized patients.

## References

[1] DHHS. The Surgeon General’s Call to Action to Prevent Deep Vein Thrombosis and Pulmonary Embolism. Office of the Surgeon General. U.S. Department of Health & Human Services Washington, DC Availabe URL (accessed August 8, 2011): http://www.surgeongeneral.gov/topics/deepvein/calltoaction/call-to-action-on-dvt-2008.pdf. 2008. 20669525

[2] Tapson VF, Decousus H, Pini M, Chong BH, Froehlich JB, Monreal M, et al Venous thromboembolism prophylaxis in acutely ill hospitalized medical patients: findings from the International Medical Prevention Registry on Venous Thromboembolism. Chest. 2007 9;132(3):936–45. 17573514 10.1378/chest.06-2993

[3] Ruppert A, Steinle T, Lees M. Economic burden of venous thromboembolism: a systematic review. Journal of medical economics. 2011;14(1):65–74. 10.3111/13696998.2010.546465 21222564

[4] Tsai J, Abe K, Boulet SL, Beckman MG, Hooper WC, Grant AM. Predictive accuracy of 29-comorbidity index for in-hospital deaths in US adult hospitalizations with a diagnosis of venous thromboembolism. PLOS ONE. 2013;8(7):e70061 10.1371/journal.pone.0070061 23922902 PMC3724730

[5] Tsai J, Grant AM, Soucie JM, Helwig A, Yusuf HR, Boulet SL, et al Clustering Patterns of Comorbidities Associated with In-Hospital Death in Hospitalizations of US Adults with Venous Thromboembolism. International journal of medical sciences. 2013;10(10):1352–60. 10.7150/ijms.6714 23983596 PMC3753416

[6] Heit JA, Melton LJ 3rd, Lohse CM, Petterson TM, Silverstein MD, Mohr DN, et al Incidence of venous thromboembolism in hospitalized patients vs community residents. Mayo Clinic proceedings Mayo Clinic. [Comparative Study Research Support, U.S. Gov't, P.H.S.]. 2001 11;76(11):1102–10. 11702898 10.4065/76.11.1102

[7] Spencer FA, Lessard D, Emery C, Reed G, Goldberg RJ. Venous thromboembolism in the outpatient setting. Arch Intern Med. [Research Support, N.I.H., Extramural]. 2007 7 23;167(14):1471–5. 17646600 10.1001/archinte.167.14.1471 PMC2762787

[8] White RH. Identifying risk factors for venous thromboembolism. Circulation. [Comment Editorial]. 2012 5 1;125(17):2051–3. 10.1161/CIRCULATIONAHA.112.102814 22474263

[9] Wolberg AS, Aleman MM, Leiderman K, Machlus KR. Procoagulant activity in hemostasis and thrombosis: Virchow's triad revisited. Anesthesia and analgesia. 2012 2;114(2):275–85. 10.1213/ANE.0b013e31823a088c 22104070 PMC3264782

[10] Chandra D, Parisini E, Mozaffarian D. Meta-analysis: travel and risk for venous thromboembolism. Ann Intern Med. 2009 8 4;151(3):180–90. 19581633 10.7326/0003-4819-151-3-200908040-00129

[11] Maynard GA, Stein J. Preventing Hospital-Acquired Venous Thromboembolism: A Guide for Effective Quality Improvement. Prepared by the Society of Hospital Medicine. AHRQ Publication No. 08–0075. Rockville, MD. AHRQ. 2010 August.

[12] Guyatt GH, Akl EA, Crowther M, Gutterman DD, Schuunemann HJ, American College of Chest Physicians Antithrombotic T, et al Executive summary: Antithrombotic Therapy and Prevention of Thrombosis, 9th ed: American College of Chest Physicians Evidence-Based Clinical Practice Guidelines. Chest. 2012 2;141(2 Suppl):7S–47S. 10.1378/chest.1412S3 22315257 PMC3278060

[13] Qaseem A, Chou R, Humphrey LL, Starkey M, Shekelle P, Clinical Guidelines Committee of the American College of P. Venous thromboembolism prophylaxis in hospitalized patients: a clinical practice guideline from the American College of Physicians. Ann Intern Med. 2011 11 1;155(9):625–32. 10.7326/0003-4819-155-9-201111010-00011 22041951

[14] Vogeli C, Kang R, Landrum M, Hasnain Wynia R, Weissman J. Quality of care provided to individual patients in US hospitals: results from an analysis of national Hospital Quality Alliance data. Medical care. 2009;47(5):591–9. 10.1097/MLR.0b013e31819432cd 19365292

[15] Foster D, Zrull L, Chenoweth J. Hospital Performance Differences by Ownership. Research Brief. 100 Top Hospitals: Study Overview, 20th Edition Ann Arbor, MI Truven Health Analytics 2013.

[16] Houchens R, Chu B, Steiner C. Hierarchical modeling using HCUP data. HCUP methods series report # 2007–01 Online. January 10, 2007. U.S. Agency for Healthcare Research and Quality. Available: http://www.hcup-us.ahrq.gov/reports/methods.jsp. Last accessed: June 14, 2013. 2007.

[17] Huseynova K, Xiong W, Ray JG, Ahmed N, Nathens AB. Venous Thromboembolism as a Marker of Quality of Care in Trauma. Journal of the American College of Surgeons. 2009 4//;208(4):547–52.e1. 10.1016/j.jamcollsurg.2009.01.002 19476788

[18] AHRQ. Overview of the Nationwide Inpatient Sample (NIS). Agency for Healthcare Research and Quality (AHRQ) Rockville, MD URL accessed on 8/2/2013: http://www.hcup-us.ahrq.gov/nisoverview.jsp. 2013.

[19] AHRQ. Introduction to the HCUP Nationwide Inpatient Sample (NIS) Healthcare Cost and Utilization Project (HCUP). 2011 Agency for Healthcare Research and Quality (AHRQ) Rockville, MD URL accessed on 8/2/2013: http://www.hcup-us.ahrq.gov/db/nation/nis/NIS_Introduction_2011.pdf. 2013.

[20] Elixhauser A, Steiner C, Harris DR, Coffey RM. Comorbidity measures for use with administrative data. Med Care. 1998 1;36(1):8–27. 9431328 10.1097/00005650-199801000-00004

[21] AHRQ. Comorbidity Software, Version 3.7 Agency for Healthcare Research and Quality (AHRQ) Rockville, MD URL accessed on 3/22/2012: http://www.hcup-us.ahrq.gov/toolssoftware/comorbidity/comorbidity.jsp. 2012.

[22] AHRQ. Procedure Classes 2012. Agency for Healthcare Research and Quality (AHRQ) Rockville, MD URL accessed on 7/12/2012: http://www.hcup-us.ahrq.gov/toolssoftware/procedure/procedure.jsp. 2012.

[23] AHRQ. Major operating room procedure indicator Agency for Healthcare Research and Quality (AHRQ) Rockville, MD URL accessed on 7/12/2012: http://www.hcup-us.ahrq.gov/db/vars/orproc/nisnote.jsp. 2012.

[24] Snijders TAB, Bosker R. Multilevel Analysis: An Introduction to Basic and Advanced Multilevel Modeling. Second ed: SAGE Publications Ltd; 2011.

[25] Carle AC. Fitting multilevel models in complex survey data with design weights: Recommendations. BMC Med Res Methodol. 2009;9:49 10.1186/1471-2288-9-49 19602263 PMC2717116

[26] Keenan CR, White RH. The effects of race/ethnicity and sex on the risk of venous thromboembolism. Current opinion in pulmonary medicine. 2007 9;13(5):377–83. 17940480 10.1097/MCP.0b013e3281eb8ef0

[27] Yusuf HR, Reyes N, Zhang QC, Okoroh EM, Siddiqi AE, Tsai J. Hospitalizations of Adults > = 60 Years of Age With Venous Thromboembolism. Clin Appl Thromb Hemost. 2013 Jun 27. 10.1177/1076029613493659 PMC4471947 23814170

[28] Piazza G, Seddighzadeh A, Goldhaber SZ. Double trouble for 2,609 hospitalized medical patients who developed deep vein thrombosis: prophylaxis omitted more often and pulmonary embolism more frequent. Chest. 2007 8;132(2):554–61. 17573518 10.1378/chest.07-0430

[29] Kaboli PJ, Brenner A, Dunn AS. Prevention of venous thromboembolism in medical and surgical patients. Cleveland Clinic journal of medicine. 2005 4;72 Suppl 1:S7–13. 15853174 10.3949/ccjm.72.suppl_1.s7

[30] Streiff MB, Lau BD. Thromboprophylaxis in nonsurgical patients. Hematology Am Soc Hematol Educ Program. 2012;2012:631–7. 10.1182/asheducation-2012.1.631 23233645

[31] Cohen AT, Alikhan R, Arcelus JI, Bergmann JF, Haas S, Merli GJ, et al Assessment of venous thromboembolism risk and the benefits of thromboprophylaxis in medical patients. Thromb Haemost. 2005 10;94(4):750–9. 16270626

[32] Dean SM, Abraham W. Venous thromboembolic disease in congestive heart failure. Congestive heart failure. 2010 Jul-Aug;16(4):164–9. 10.1111/j.1751-7133.2010.00148.x 20662869

[33] Lyman GH. Venous thromboembolism in the patient with cancer: focus on burden of disease and benefits of thromboprophylaxis. Cancer. 2011 4 1;117(7):1334–49. 10.1002/cncr.25714 21425133 PMC3780385

[34] Lopes AA. Pathophysiological basis for anticoagulant and antithrombotic therapy in pulmonary hypertension. Cardiovascular & hematological agents in medicinal chemistry. 2006 1;4(1):53–9. 16529549 10.2174/187152506775268794

[35] Ageno W, Becattini C, Brighton T, Selby R, Kamphuisen PW. Cardiovascular risk factors and venous thromboembolism: a meta-analysis. Circulation. 2008 1 1;117(1):93–102. 18086925 10.1161/CIRCULATIONAHA.107.709204

[36] Kim SC, Schneeweiss S, Liu J, Solomon DH. Risk of venous thromboembolism in patients with rheumatoid arthritis. Arthritis care & research. 2013 10;65(10):1600–7. 23666917 10.1002/acr.22039 PMC4090802

[37] Tveit DP, Hypolite IO, Hshieh P, Cruess D, Agodoa LY, Welch PG, et al Chronic dialysis patients have high risk for pulmonary embolism. American journal of kidney diseases: the official journal of the National Kidney Foundation. 2002 5;39(5):1011–7. 11979344 10.1053/ajkd.2002.32774

[38] Heit JA, Silverstein MD, Mohr DN, Petterson TM, O'Fallon WM, Melton LJ 3rd. Risk factors for deep vein thrombosis and pulmonary embolism: a population-based case-control study. Arch Intern Med. 2000 3 27;160(6):809–15. 10737280 10.1001/archinte.160.6.809

[39] Dabbagh O, Oza A, Prakash S, Sunna R, Saettele TM. Coagulopathy does not protect against venous thromboembolism in hospitalized patients with chronic liver disease. Chest. 2010 5;137(5):1145–9. 10.1378/chest.09-2177 20040609

[40] Bibas M, Biava G, Antinori A. HIV-Associated Venous Thromboembolism. Mediterranean journal of hematology and infectious diseases. 2011;3(1):e2011030 10.4084/MJHID.2011.030 21869916 PMC3152452

[41] Franchini M, Targher G, Montagnana M, Lippi G. Iron and thrombosis. Annals of hematology. 2008 3;87(3):167–73. 18066546 10.1007/s00277-007-0416-1 PMC2226003

[42] Sundstrom A, Seaman H, Kieler H, Alfredsson L. The risk of venous thromboembolism associated with the use of tranexamic acid and other drugs used to treat menorrhagia: a case-control study using the General Practice Research Database. BJOG. 2009 1;116(1):91–7. 10.1111/j.1471-0528.2008.01926.x 19016686

[43] Wattanakit K, Cushman M, Stehman-Breen C, Heckbert SR, Folsom AR. Chronic kidney disease increases risk for venous thromboembolism. Journal of the American Society of Nephrology: JASN. 2008 1;19(1):135–40. 18032796 10.1681/ASN.2007030308 PMC2391038

[44] Folsom AR, Boland LL, Cushman M, Heckbert SR, Rosamond WD, Walston JD. Frailty and risk of venous thromboembolism in older adults. The journals of gerontology Series A, Biological sciences and medical sciences. 2007 1;62(1):79–82. 17301042 10.1093/gerona/62.1.79

[45] Valderas JM, Starfield B, Sibbald B, Salisbury C, Roland M. Defining comorbidity: implications for understanding health and health services. Annals of family medicine. [Research Support, Non-U.S. Gov't Review]. 2009 Jul-Aug;7(4):357–63. 10.1370/afm.983 19597174 PMC2713155

[46] Pickett KE, Pearl M. Multilevel analyses of neighbourhood socioeconomic context and health outcomes: a critical review. J Epidemiol Community Health. 2001 2;55(2):111–22. 11154250 10.1136/jech.55.2.111 PMC1731829

[47] Papanikolaou PN, Christidi GD, Ioannidis JP. Patient outcomes with teaching versus nonteaching healthcare: a systematic review. PLoS Med. 2006 9;3(9):e341 16968119 10.1371/journal.pmed.0030341 PMC1564172

[48] Thornlow D, Stukenborg G. The association between hospital characteristics and rates of preventable complications and adverse events. Medical care. 2006;44(3):265–9. 16501398 10.1097/01.mlr.0000199668.42261.a3

[49] Sarrazin MS, Rosenthal GE. Finding pure and simple truths with administrative data. JAMA. [Comment Editorial]. 2012 4 4;307(13):1433–5. 10.1001/jama.2012.404 22474208

[50] Duncan C, Jones K, Moon G. Context, composition and heterogeneity: using multilevel models in health research. Soc Sci Med. 1998 1;46(1):97–117. 9464672 10.1016/s0277-9536(97)00148-2

